# The role of anti-aging approaches in managing hypogonadism in sedentary older males

**DOI:** 10.3389/fragi.2024.1514438

**Published:** 2024-11-13

**Authors:** Khaled A. Abdel-Sater

**Affiliations:** Faculty of Dentistry, Mutah University, Karak, Jordan

**Keywords:** sedentary life, antiaging strategies, testosterone, testosterone replacement therapy, elderly men, hypogonadism

## Abstract

**Introduction:**

With thirty percent of the world’s population not getting enough exercise, Worldwide, physical inactivity ranks as one of the most common causes of premature mortality. Rapid drops in physical activity, decreased mobility, and early morbidity are aging characteristics. As the population over 80 continues to rise, aging raises the danger of age-related illnesses and changes in hormone release.

**Aim:**

Understanding the aging process is useful in developing pharmacological therapies and identifying therapeutic targets for age-related testosterone deficiency. Therefore, this study’s purpose is to present a thorough evaluation of the effects of anti-aging strategies on testosterone levels in older, inactive men.

**Methods:**

A literature search was completed for clinical and preclinical studies published in English between 2014 and 2024 related to age, sedentary life, testosterone, and anti-aging strategies.

**Results:**

A sedentary lifestyle and low testosterone are linked to a vicious cycle. A sedentary lifestyle lowers testosterone levels, which leads to depression, exhaustion, low energy, and weakened bone and muscle strength. These effects exacerbate the detrimental consequences of aging and physical inactivity. Anti-aging techniques can prevent and treat age-related diseases, including calorie restriction, a balanced diet, regular exercise, weight control, diabetes management, and quitting smoking. Regular exercise raises total testosterone, free testosterone, and muscle steroidogenesis. In older men, testosterone replacement treatment increases bone density, cholesterol, protein synthesis, strength, erectile function, sexual desire, and general cognitive performance. However, some studies suggest dehydroepiandrosterone supplementation may provide health improvements without negative effects, potentially reversing arterial aging and reducing cardiovascular disease risk.

**Conclusion:**

This article evaluates the prospects for anti-aging procedures to assist in reducing the adverse effects of aging and physical inactivity in men.

## Introduction

Worldwide, physical inactivity ranks as one of the most common causes of premature mortality (6% of deaths globally) (WHO, 2020). According to the World Health Organization’s (WHO) estimates, 31% of people worldwide do not engage in regular exercise to maintain their health (Guthold et al., 2018). Women are generally less active than males, and there are notable regional and national variations in physical activity levels. It is greatest in nations with high incomes. Owing to shifting mobility patterns, rising technological use, and urbanization, inactivity rates in some nations can reach 70% (Altenburg et al., 2017). There is a positive correlation between physical inactivity and chronic illnesses, non-communicable diseases, and early death (Katzmarzyk et al., 2022). According to estimates, physical inactivity contributes between 6% and 10% of premature deaths worldwide, 30% of instances of coronary heart disease, 27% of type 2 diabetes, and obesity, and between 21% and 25% of cases of breast and colon cancer (Ding et al., 2017). The proportion of people 60 years of age and older is increasing. 1.4 billion people in the world will be 60 years of age or older by 2030, and by 2050 that number will rise to 2.1 billion (WHO, 2022).

Aging increases the risks of many illnesses, such as diabetes, heart diseases, cataracts, arthritis, osteoporosis, benign prostatic hyperplasia, stroke, chronic obstructive pulmonary disease, cancer, and neurodegenerative and endocrinal diseases (Li et al., 2021).

Elder men are at risk of sedentary lifestyles. Understanding the aging process will hopefully help in finding therapeutic targets for age-related testosterone deficiency and in drug discovery for future authorized clinical uses (Zhao et al., 2021). Therefore, this review aims to provide a systematic overview of the effect of antiaging strategies on testosterone in sedentary older males.

## The decline of testosterone with age

Testosterone levels in men begin to decline at around 30–40 years of age and continue until death (Zhu et al., 2022). Annually the level of testosterone decreases at a rate of around 2.8% (Erenpreiss et al., 2020). Twenty percent of men in their 60s and over fifty percent of men in their 80s had serum testosterone levels considerably subnormal (Zhu et al., 2022).

Local and systemic illness, prescription medications, lung tumors, excessive smoking or alcohol, obesity, and untreated diabetes were correlated with a decrease in testosterone (Wittert and Grossmann, 2022). Testosterone levels have been reported in untreated elderly diabetic males approximately 15% lower compared to their non-diabetic counterparts (Hasan et al., 2022). Opioids and anticonvulsants can negatively impact testosterone production by altering hormonal signaling or enzyme activity. Opioids change the luteinizing hormone (LH) pulsatility while anticonvulsants decrease the hepatic enzyme induction (Platz et al., 2019). Regardless of age, both smoking and lung tumors lower testosterone levels (Koh et al., 2022). According to Okwara et al. (2019), chronic alcohol users had testosterone levels that were 12% lower than those of non-drinkers. Low testosterone levels are strongly linked to male obesity and diabetes. Reduced levels of testosterone are closely associated with several variables, including visceral obesity, insulin resistance, poor glycemic control, and longer duration of diabetes (Khalil et al., 2024).

There are many factors involved in the reduction of testosterone levels with aging. These include a decrease in the number of Leydig cells and their responsiveness (Leydig cell aging), as well as a decrease in the pituitary luteinizing hormone and gonadotropin-releasing hormone releases (Wittert and Grossmann, 2022). Aging is associated with a dampening of the diurnal rhythm of testosterone secretion with a rise in the levels of sex hormone-binding globulins. This rise in sex hormone-binding globulin levels is caused by a rise in manufacture (Yeap et al., 2016).

The reduction of testosterone level is associated with cardiovascular (such as hypertension and ischemia), metabolic (such as metabolic syndrome, insulin resistance, and type 2 diabetes) (Wittert and Grossmann, 2022), psychological (such as depression, irritability, fatigue, difficulty concentrating and decreased energy level) (Zhu et al., 2022), physical (such as deteriorated muscle and bone mass), and sexual (such as impotence, erectile dysfunction, and decreased libido) features (Ramachandran et al., 2024).

## The decline of testosterone with an inactive lifestyle

Sedentary lifestyles significantly exacerbate men’s natural decline in testosterone as they age. Because of decreased blood flow, excessive body fat, muscle loss, elevated stress, and disturbed sleep, a sedentary lifestyle can greatly contribute to a drop in testosterone levels (Chasland et al., 2021).

Inactivity is associated with metabolic syndrome, weight gain, and increased fat mass (specifically visceral fat) that can convert testosterone into estrogen, further lowering the hormone levels. Visceral fat secretes hormones and inflammatory agents (Riachy et al., 2020). Chronic inflammation triggered by excess fat disrupts the signaling processes involved in testosterone synthesis, directly suppressing its production. Also, these factors, in turn, negatively affect the hypothalamus pituitary adrenal axis, leading to further reductions in testosterone levels (Chasland et al., 2021).

Without regular exercise, there is a loss of muscle mass. This can decrease testosterone production, as muscle mass plays a vital role in hormone synthesis (Schwanbeck et al., 2020). Sedentary lifestyles also increase the risk of metabolic syndrome, which causes a decline in testosterone (Riachy et al., 2020). Higher stress levels and disrupted sleep can disrupt hormone balance and lower testosterone. Cortisol directly antagonizes testosterone production (Liu and Reddy, 2022).

So, there is a vicious cycle between low testosterone and a sedentary lifestyle. A sedentary lifestyle causes a decline in testosterone levels that causes depression, fatigue, decreased energy levels, deteriorated muscle and bone mass, difficulty concentrating, and heart disease. These effects further compound the negative impacts of aging and physical inactivity. This cycle exacerbates health outcomes.

### Antiaging strategies

These strategies can encompass a range of lifestyle modifications, medical therapies, and stem Leydig cell transplantation.

### Lifestyle modifications

They include physical activity, a balanced diet, and healthy sleep. It has been demonstrated that maintaining a normal weight and exercise level can safely raise testosterone secretion. A normal body weight reduces the risk of low testosterone levels associated with obesity (Mulhall et al., 2018).

#### A- physical activity

The most effective non-pharmacological method for raising testosterone production is regular exercise (Chasland et al., 2021). The level of testosterone depends upon type (i.e., resistance or endurance), frequency, volume, intensity (high, moderate, and low), and duration of exercise (WHO, 2020). The WHO physical activity guidelines advise people to exercise for at least 150–300 min a week at a moderate to intense level 75–150 min a week at a vigorous level, or an equivalent combination (Bull et al., 2020). It could be necessary to engage in greater activity if one wants to maintain or lose weight (Ilić et al., 2022).

Physical activity improves the following health outcomes: decreased anxiety and depressive symptoms, better sleep, increased mortality from cardiovascular disease and other causes, incident hypertension, incident cancers specific to specific sites, incident type-2 diabetes, and improved measures of adiposity (Mascarenhas et al., 2024). Engaging in physical activity assists elderly individuals in preventing falls, the harm they cause, and the decline in their bone density and functional capacity (Bull et al., 2020).

Acute endurance exercise (such as running, cycling, or swimming) increases the testosterone level while chronic high-volume endurance exercise may lead to lower testosterone levels (Mascarenhas et al., 2024). Strength training or weightlifting is generally associated with increases in testosterone levels (Guthold et al., 2018). Regular, consistent training (as opposed to sporadic sessions) tends to lead to better hormonal responses (Bull et al., 2020). However, overtraining or inadequate recovery can suppress testosterone levels (Chasland et al., 2021). The amount of muscle involved in resistance exercises affects testosterone response. Larger muscle groups (e.g., legs and back) tend to elicit a greater hormonal response than smaller muscle groups. Higher volume (more sets and repetitions) often results in greater acute increases in testosterone (Ilić et al., 2022). Increases in testosterone are often observed after high-intensity resistance training sessions. Short-duration, high-intensity workouts typically lead to acute increases in testosterone (Guthold et al., 2018). However, prolonged exercise (especially at high volumes in endurance sports) can result in a decrease in testosterone levels due to increased cortisol production and energy deficit (Riachy et al., 2020).

Chasland et al. (2021) stated that physical activity was more effective than testosterone treatment in increasing muscular strength and decreasing total and visceral fat mass. Testosterone treatment at therapeutic doses increased lean mass but conferred limited additional benefit when combined with exercise.

#### B- sleep

Prioritizing sleep is essential for physical and mental restoration. Chronic sleep deprivation can accelerate aging and is associated with a decline in testosterone levels (Chasland et al., 2021). The symptoms of testosterone insufficiency and self-reported sleep quality are correlated linearly (Armamento-Villareal et al., 2016).

#### C- stress management

Chronic stress is likely to lower testosterone levels due to the direct inhibition of cortisol on Leydig cells and the hypothalamic-pituitary-gonadal axis (Armamento-Villareal et al., 2016). Reducing stress through techniques like mindfulness, yoga, or meditation can lower cortisol levels and elevate testosterone levels (Banihani, 2019).

#### D- nutrition

Consuming a balanced diet composed of an abundance of healthful grains, fruits, veggies, lean meats, and healthy fats can help supply essential nutrients essential for testosterone production and combat oxidative stress and inflammation (Liu and Reddy, 2022). Total testosterone levels significantly increased in response to a weight-loss diet, both with and without frequent exercise (Wittert and Grossmann, 2022). When honey is taken orally, it raises men’s blood testosterone levels by stimulating the synthesis of luteinizing hormone (Armamento-Villareal et al., 2016). It is packed with antioxidants, minerals (such as zinc), and many vitamins (including B vitamins). Particularly zinc is recognized to be essential for the synthesis of testosterone (Banihani, 2019).

#### Caloric restriction (CR)

CR is reducing caloric intake by 20%–60% without malnutrition. It delays age-related disorders and can increase the lifespan of mice by as much as 40% or more. Numerous factors, including age, sex, health status, and degree of CR influence its impact on testosterone levels. Some studies suggest that short-term caloric restriction can lead to a decline in testosterone levels. This is because of the body’s adaptive response to reduced energy availability, where it conserves energy and minimizes reproductive functions, including testosterone production (Dorling et al., 2021).

Long-term calorie restriction frequently results in weight loss, and in overweight or obese people in particular, a decrease in body fat may have a favorable impact on testosterone levels. Excess visceral fat is associated with lower testosterone levels, so losing fat can help restore or improve hormonal balance (Wittert and Grossmann, 2022). This happens through two different mechanisms: enhanced testicular function and decreased testosterone conversion to β-estradiol via adipose tissue’s aromatase activity (Wiciński et al., 2023).

#### CR mimetics

CR mimetics are compounds that aim to mimic the physiological effects of calorie restriction without actually reducing caloric intake. The CR mimetics include natural compounds (such as resveratrol, curcumin, and quercetin), and pharmacological drugs (such as metformin and rapamycin) (Zhu et al., 2018).

A significant natural polyphenolic chemical, resveratrol may be found in several fruits and vegetables, including peanuts, grapes, and peanut sprouts. It is a sirtuin-activating compound that has been shown to extend lifespan and modulate insulin secretion and action (Zhu et al., 2023). Resveratrol effectively inhibits several processes, including angiogenesis, oxidative stress, apoptosis, inflammation, and mitochondrial malfunction. Furthermore, it inhibits platelet aggregation to further exhibit its cardioprotective effects (Hassanin et al., 2024). It has been demonstrated that resveratrol supplementation causes a penile erection, reduces germ cell apoptosis, increases blood testosterone concentration, and enhances sperm quality and epididymal sperm count (Belhan et al., 2020). The hypothalamic-pituitary-gonadal axis is directly stimulated by resveratrol while the testes are unaffected (Bidian et al., 2020). On the other hand, Semba et al. (2014) stated that the dietary resveratrol from Western diets did not significantly affect lifespan in older persons living in the community. Its effects require adjusting for the ideal dose and possible drug interactions. It will remain important to do more human research on the framework for using resveratrol as an anti-aging medication.

Curcumin, derived from turmeric, curcumin has antioxidant, anti-inflammatory, and antiapoptotic properties. It enhanced testicular hemodynamics, follicle-stimulating hormone, testosterone, and nitric oxide levels while markedly suppressing the lipid profile (Wang et al., 2022). On the other hand, Strong et al. (2013) stated that the log-rank test revealed that neither resveratrol nor curcumin significantly affected the lifespan of either male or female mice.

Quercetin, present in vegetables and fruits may mimic some effects of CR and promote health span (Pastuszak et al., 2017). It has been shown that quercetin inhibits the enzyme that converts testosterone into testosterone glucuronide, enhancing testicular functioning and testosterone levels (Ullah et al., 2021).

### Medical therapies

Testosterone replacement therapy in old man led to advancements in physical function, strength, protein synthesis, cholesterol, and bone density (Zhu et al., 2022). The testosterone trials (T-Trials) and testosterone optimization and monitoring trials (TOM-Trials) are two important studies that investigated the effects of testosterone replacement therapy in men. T-Trials focus on sexual function, bone density, muscle mass and strength, cognitive function, and cardiovascular health. They provided insights into the benefits of testosterone therapy, including improvements in sexual function, increased muscle mass, and enhanced overall quality of life. The TOM-Trials aimed to provide a more comprehensive understanding of testosterone therapy in different demographic groups and contribute to establishing guidelines for optimal monitoring and treatment strategies for men receiving testosterone replacement therapy (Snyder et al., 2018).

Ramachandran et al. (2024) stated that testosterone replacement therapy is associated with improved the aging male symptom scale (cardiovascular, metabolic, psychological, physical, and sexual features) in men with T2DM and adult-onset testosterone deficiency demonstrating symptoms with this benefit mediated by levels of depression and anxiety. Administering testosterone elevates the expression of glucose transporter type 4 and enhances the activity of glycolytic enzymes (hexokinase, phosphofructokinase, and glycogen synthase) (Erenpreiss et al., 2020).

Green et al. (2024) stated that testosterone has impacts on strength, body composition, and aerobic fitness outcomes that are dependent upon dose, route of administration, and formulation.

However, not every elderly man will benefit from this replacement. According to some studies, testosterone replacement therapy for older men does not significantly improve strength or cognitive function, but it does cause venous thromboembolism, heart attack, and stroke. It also causes exacerbated symptoms of both benign prostatic hyperplasia and polycythemia. Regular monitoring of testosterone levels, as well as screenings for prostate health, edema, heart, and renal functions, is crucial during replacement (Zitzmann, 2024).

While the effectiveness of testosterone replacement therapy is still debatable, some have hypothesized that administering the testosterone precursor dehydroepiandrosterone (DHEA) might enhance health with minor adverse consequences (Pataky et al., 2021).

Male hypogonadism can be effectively treated using gonadotropins, such as human chorionic gonadotropin (hCG) (approved by the FDA for this purpose). However, gonadotropins are expensive and must be administered by intramuscular or subcutaneous injection. hCG has the same structure and function as the LH, and their receptors are identical (Ilić et al., 2022). It is both a safer and more efficient treatment and can be used in patients with pituitary dysfunction. It stimulates the testes to produce more testosterone and is particularly useful for men desiring to preserve spermatogenesis and fertility. It has potential side effects such as gynecomastia, breathing problems, emotional instability, and testicular swelling (Fink et al., 2024).

Selective estrogen receptor modulators (SERMs) enhance the synthesis of LH and subsequent testosterone by blocking the negative feedback that estrogen has on the hypothalamus. It is used to restore testosterone levels while preserving fertility by stimulating the body’s testosterone production. Long-term usage of SERMs seems to be safe and well-tolerated, but venous thromboembolism, joint swelling, and gastrointestinal discomfort may occur (Handrapal et al., 2016).

Aromatase inhibitors can help increase testosterone levels by preventing the conversion of testosterone to estrogen, thus enhancing endogenous testosterone production. These drugs may also increase testosterone levels, but if used over an extended period, the suppression of estrogen may result in decreased bone mineral density. They are also designed to prevent the tissues from converting testosterone into estrogen, which suppresses the hypothalamic-pituitary-gonadal axis in the presence of estrogen. While aromatase inhibitors can be effective, they may also have side effects such as bone density loss, mood swings, and weight gain (Dias et al., 2017).

It also was found that nerve growth factor increased LH, FSH, and testosterone levels as well as indices of sexual behavior in mice, such as mounting and ejaculation times (Luo et al., 2018).

It has also been demonstrated that oxytocin stimulates the production of GnRH, and it has been suggested that intranasal oxytocin might 1 day be used as a treatment for testosterone insufficiency (Salehi et al., 2017).

Senolytics, including dasatinib, fisetin, and navitoclax, improved sperm concentration and serum testosterone levels while decreasing aberrant sperm morphology; they did not affect fertility. The antioxidant impact is what causes this result (Garcia et al., 2023).

Vitamin D insufficiency in the elderly is associated with a reduction of muscle mass and function, Osteoporosis diminished cognitive function, and a significant decrease in rat testis and spermatogenesis (Kupisz-Urbańska et al., 2021). Reduced androgen production is at least partially responsible for these findings (Zamani et al., 2020).

### Stem Leydig cell transplantation

It can undergo differentiation into fully developed Leydig cells, be subject to hypothalamic-pituitary-gonadal axis regulation, and reestablish circadian rhythm testosterone production. Most of the current research on stem Leydig cell transplantation has been conducted in animal models (Zang et al., 2017). More research is needed to translate these findings into clinical applications for humans. Factors such as the source of stem cells, transplantation techniques, and the long-term effects of such interventions are still under investigation (Hoang et al., 2022).

## Conclusion

The prevention and treatment of aging-related testosterone loss is promising but challenging. Anti-aging strategies include lifestyle modifications - such as physical activity, a balanced diet, and restful sleep- and medical therapies to increase testosterone production. Moderate exercise is the most effective non-pharmacological method for raising testosterone production, while a balanced diet can provide essential nutrients for testosterone production and combat oxidative stress and inflammation. CR can delay aging and increase lifespan, while natural compounds like resveratrol, curcumin, and quercetin can also help improve testicular function and testosterone levels. Medical therapies like testosterone replacement therapy, gonadotropins, SERMs, aromatase inhibitors, nerve growth factors, and oxytocin may help restore testosterone levels.
